# Head and Neck Squamous Cell Carcinoma: Epigenetic Landscape

**DOI:** 10.3390/diagnostics11010034

**Published:** 2020-12-27

**Authors:** Kamila Romanowska, Agnieszka Sobecka, Agnieszka A. Rawłuszko-Wieczorek, Wiktoria M. Suchorska, Wojciech Golusiński

**Affiliations:** 1 Department of Head and Neck Surgery, Poznan University of Medical Sciences, The Greater Poland Cancer Centre, 61-866 Poznan, Poland; agnieszka.sobecka@wco.pl (A.S.); wgolus@ump.edu.pl (W.G.); 2 Department of Medical Physics, Radiobiology Laboratory, Poznan University of Medical Sciences, The Greater Poland Cancer Centre, 61-866 Poznan, Poland; wiktoria.suchorska@wco.pl; 3 Department of Histology and Embryology, Poznan University of Medical Sciences, 60-781 Poznan, Poland; arawluszko@ump.edu.pl

**Keywords:** head and neck cancer, squamous cell carcinoma, epigenetics, DNA methylation, histone modification, non-coding RNA activity, RNA methylation, biomarkers

## Abstract

Head and neck squamous carcinoma (HNSCC) constitutes the sixth most prevalent cancer worldwide. The molecular pathogenesis of HNSCC includes disorders in cell cycle, intercellular signaling, proliferation, squamous cell differentiation and apoptosis. In addition to the genetic mutations, changes in HNSCC are also characterized by the accumulation of epigenetic alterations such as DNA methylation, histone modifications, non-coding RNA activity and RNA methylation. In fact, some of them may promote cancer formation and progression by controlling the gene expression machinery, hence, they could be used as biomarkers in the clinical surveillance of HNSCC or as targets for therapeutic strategies. In this review, we focus on the current knowledge regarding epigenetic modifications observed in HNSCC and its predictive value for cancer development.

## 1. Introduction

Head and neck squamous cell carcinoma (HNSCC) is a common heterogeneous malignant cancer type originating from the squamous cells, located in the mucous membrane of the oral cavity, oropharynx, paranasal sinuses, nasal cavity, nasopharynx, larynx and hypopharynx [1]. The main prognostic features of HNSCC progression are the location, tumor size and the presence of distant metastases [2]. The estimated number of HNSCC accounts for more than 650,000 cases and 330,000 deaths annually [3]. Main and widely investigated contributors to the development of head and neck cancers are tobacco smoking, alcohol consumption as well as viral factors such as human papillomavirus (HPV) and Epstein–Barr virus infections [4,5,6,7]. The treatment of an HNSCC patient involves surgical eradication, radiotherapy (RT) and chemotherapy (CT). Moreover, the approved targeted drug is cetuximab, a monoclonal antibody targeting epidermal growth factor receptor (EGFR) for both HPV(+) and HPV(–) subtypes [8]. The treatment method depends on the type and stage of cancer, possible side effects and the patient’s overall health. Unfortunately, cetuximab and other therapies have a limited efficacy due to molecular and histological diversity of HNSCC [9]. 

Molecular pathogenesis of HNSCC is a complex process with a high rate of genetic heterogeneity. It is possible to distinguish alterations in the tumor suppressor pathways p53, p16^INK4a^ and retinoblastoma (RB) which affects DNA damage response, apoptosis, cell cycle and genomic stability [10,11,12,13]. Additionally, overexpression of *EGFR* correlates with poor prognosis and metastatic potential of cancer cells [14]. Moreover, disorders in signaling pathways associated with Ras-mitogen activated protein kinase (Ras-MAPK) lead to disturbances in gene expression level involved in the cell proliferation, differentiation, apoptosis, angiogenesis and cell motility. Similarly, a higher rate of cancer recurrence and metastases is associated with mutations in *NOTCH1-4* genes in HNSCC [15]. Moreover, PI3K-Akt/mTOR constitutes a frequently disturbed pathway in HNSCC and simultaneously is a cascade responsible for phosphorylation and activation of many proteins [16]. The activation of STAT3 pathway, crucial in many cancer types including HNSCC, leads to a malignant transformation of cells and protect them from recognition and degradation by cytotoxic T lymphocytes [17]. Furthermore, overexpression of hepatocyte growth factor receptor *(MET)* is correlated with cisplatin and EGFR-targeted therapies resistance as well as with poor prognosis for HNSCC patients [18]. Additionally, a nuclear transcription factor-κB (NF-κB) modulating the expression of genes involved in inflammation, immunity, proliferation, and apoptosis is constitutively activated in HNSCC, and affects the therapeutic resistance [19]. All these signaling cascades shape complex cellular conditions which ultimately affect squamous epithelial proliferation and differentiation, cell survival and metastatic phenotype.

It is crucial to bear in mind the fact that, carcinogenesis of HNSCC is driven not only by the accumulation of genetic alterations, but also by the changes in the epigenetic landscape. Epigenetic modifications found in HNSCC include DNA methylation, histone modification, non-coding RNA activity, as well as RNA methylation [20,21]. Since these modifications regulate the expression of target genes (tumor suppressor genes (TSGs) and oncogenes), they have become a focus of attention in cancer studies, also in terms of personalized therapy strategies. They may be involved in the pathology of the disease; therefore, they are considered candidates for diagnostic biomarkers and prognostic features of cancer. In this review, we discuss the current literature associated with the impact of epigenetic modification on the progression of head and neck squamous cell carcinoma.

## 2. The HNSCC Epigenetic Landscape and Its Clinical Implications

### 2.1. DNA Methylation

DNA methylation is one of the best investigated DNA modifications which modulates the expression of genes without affecting their nucleotide sequence. DNA methylation is a process of covalent conversion of a hydrogen atom into a methyl group at the fifth carbon of the pyrimidine ring of cytosine (5-methylcytosine, 5-mC). In fact, this modification constitutes an essential epigenetic marker recognized by specific proteins. 

In mammals, 5-mC is highly accumulated in the DNA regions rich in CpG dinucleotides (so-called CpG islands) where 70–80% of cytosines are methylated [22]. About 60% of CpG islands are located in the gene promoter regions [23]. The presence of DNA methylation in promoters causes transcriptional repression by preventing the binding of transcription factors and by influencing interactions between enhancers and promoters [24]. Furthermore, 5-mCs are also found in repetitive sequences, gene bodies and intergenic regions. 9i [25,26]. DNA methylation is also found in non-CpG sites and it includes methylation at cytosine followed by adenine (CpA), thymine, (CpT) or another cytosine (CpC) [27]. On the other hand, non-CpG methylation is tissue-specific and functions as a transcriptional repressor by blocking transcription factors binding sites [28]. 

The enzymes responsible for DNA methylation belong to the DNA methyltransferase (DNMT) family: DNMT1, DNMT2, DNMT3A, DNMT3B and DNMT3L [29]. In fact, DNMT1 is responsible for maintaining methylation patterns after replication [30], whereas DNMT2 is a methyltransferase homologue which mainly methylates aspartic acid cytosine 38 in the tRNA anti-codon loop [31,32]. Recent reports have indicated that DNMT2 can also methylate other RNA molecules and short DNA segments in vitro [33,34]. Moreover, DNMT3A and DNMT3B are responsible for de novo DNA methylation and are particularly crucial in the embryonic development during determining methylation pattern. Last but not least, DNMT3L lacks catalytic activity, but supports DNMT3A/B in the binding of the methyl donor group S-adenosylmethionine (SAM) and regulates their multimerization and nuclear localization [35]. 

DNA methylation is a reversible modification which may occur as a passive or active mechanism. Passive DNA demethylation occurs by inhibition or lack of DNMTs activity, during DNA replication [35]. In contrast, active DNA demethylation is mediated by specific enzymes from the TET (Tet methylcytosine dioxygenase) family, regardless of DNA replication [36]. TET oxidize 5-methylcytosine to 5-hydroxymethylcytosine (5-hmC), 5-formylcytosine (5-fC) or 5-carboxylcytosine (5caC) with an altered preference. Furthermore, at least four mechanisms of demethylation to cytosine have been proposed [37]. The first mechanism suggests a replication-dependent passive dilution of 5-mC, whereas the second one includes an active replication-independent demethylation based on 5-mC removing to cytosine by thymine-DNA glycosylase (TDG) in the base excision repair (BER) mechanism. Another process is based on enzymatic 5-caC decarboxylation to cytosine and the last one is associated with the activation-induced deaminase/apolipoprotein B mRNA-editing enzyme complex (AID/APOBEC), which can deaminate 5-hmC to 5-hmU [38,39]. 

One of the cancer hallmarks is a global DNA hypomethylation and specific local hypermethylation of CpG islands [38]. Hypomethylation in cancerous tissues appears predominantly on multiple repeats elements (e.g., SAT2) and retrotransposons (e.g., LINE-1 and ALU) sequences leading to genomic instability and activation of oncogenes. Local hypermethylation of DNA is usually associated with CpG islands in promoters of tumor suppressor genes where their expression is downregulated [39]. In HNSCC patients, numerous aberrantly methylated genes have been identified. The altered methylation patterns of the selected genes have been correlated with HNSCC formation and progression, based on clinical data, and proposed as a potential biomarker of the disease progression with specific diagnostic significance (see Table 1). Unfortunately, there is a lack of research concerning its diagnostic potential in in vitro and/or in vivo assays.

In addition, the genome-wide DNA methylation assays also possess significant predictive and diagnostic value. According to the literature, whole-genome analysis of DNA methylation has been carried out in the peripheral blood of HNSCC patients in which differently methylated CpG sites have been identified in comparison to controls [53,54,55,56]. This non-invasive approach allows to identify global specifically methylated (hypo- or hypermethylated) regions of the DNA, particularly within promoters of genes. Moreover, the array-based DNA methylation profiling of HNSCC allows to distinguish tumors in terms of environmental factors and contributes to a personalized therapy [57]. Additionally, there are differences in DNA methylation profiles between HPV(+) and HPV(–) HNSCC as shown on the whole-genome sequencing data. The HPV(+) tumors tend to be more globally methylated than HPV(–) [58]. Nevertheless, the novel promising non-invasive prognostic tool for HPV(+) are biomarkers, such as circulating tumor DNA (ctDNA) from blood. In fact, the DNA methylation of *CALML5*, *DNAJC5G* and *LY6D* genes found in ctDNA from HNSCC patients had high predictive value in early diagnosis [59].

### 2.2. Histone Modifications 

Histone proteins undergo many different post-translational modifications such as acetylation, methylation, phosphorylation, ubiquitination or sumoylation which leads to global epigenetic alterations in cancer cells [60]. However, the most described mechanisms with prognostic potential for HNSCC development and progression include histone acetylation and methylation [61].

**Figure 1:**
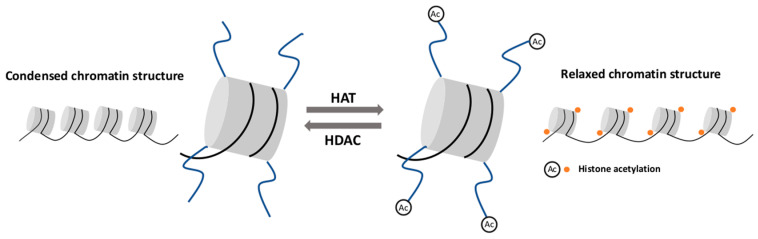
Histone acetylation and deacetylation. Histone acetyltransferase (HAT) attaches the acetyl group to the histone tail and leads to chromatin structure relaxation. Histone deacetylase (HDAC) removes the acetyl group and causes chromatin condensation.

Histone acetylation is an important mechanism affecting the chromatin structure and regulating gene expression [62,63]. Histone acetyltransferase (HAT) is the enzyme responsible for attaching the acetyl group to a specific lysine residue, mostly on H3 and H4 histone [64]. Histone acetylation neutralizes the positive charge of lysine residues and relaxes the chromatin structure. This process is correlated with the recruitment of transcription coactivators and an increased transcription elongation performed by RNA polymerase II [62]. In principle, histone deacetylase (HDAC) is responsible for the removal of acetyl groups, restoring a positive charge to lysine residues and consequently, leading to chromatin condensation. This configuration limits the availability of DNA for transcription factors and results in transcriptional inhibition [65] (Figure 1). 

One of the characteristic factors of the solid and metastatic tumor as HNSCC is hypoxic microenvironment [66]. In response to hypoxia, H3K2 is acetylated and activates the epithelial mesenchymal transition (EMT) correlated genes, including *GLI1* and *SMO* genes, thus increasing the metastatic potential of the tumor. In fact, these genes may be considered as hypoxia-induced EMT biomarkers of HNSCC [67]. Furthermore, in oral squamous carcinoma (OSCC), acetylation of H3K27 increased the expression of long non-coding RNA (lncRNA) PLAC2, which induced Wnt/β-catenin signaling cascade and affected tumor growth and metastases. Hence, overexpression of PLAC2 may be a prognostic biomarker of metastases in OSCC [68]. Moreover, poor prognosis of the HNSCC patients may stem from chemoresistance. The overexpression of NF-κB protein complex leads both to histone deacetylation and to cisplatin resistance by means of reducing BRCA1 nuclear translocation in HNSCC. Therefore, NF-κB protein complex would constitute as chemoresistance biomarker for HNSCC [69]. Additionally, in vitro HNSCC cells assay shows global histone H3 hypoacetylation as compared to normal oral keratinocytes. Moreover, inhibition of HDAC leads to decreased number of cancer stem cells (CSC) and reduces the clonogenic sphere formation [70]. Interestingly, HDAC inhibitors also possess the ability to inactivate other genes such as ARF1 which affects the EGFR degradation and the inhibition of HNSCC cells invasion [71].

Histone methylation in a lysine (Lys or K) or arginine (Arg or R) residue constitutes another posttranslational modification which plays a vital role in gene regulation. These modifications can be recognized by multiple positive and negative regulators activating or repressing gene transcription [72]. According to the literature, lysine residues in histone can be mono-, di-, or tri-methylated. Di- and tri-methylation at H3K4, H3K36 and H3K79 are typically gene-activating, whereas H3K9 and H3K27 methylations are generally gene-repressive [73]. Moreover, H3K4me3 marks promoters, as well as H3K36 and H3K79 methylation occurs primarily over gene bodies [72,74]. Histone methyltransferases (HMT) includes histone lysine methyltransferases (HKMT) and protein/histone arginine methyltransferases (PRMT) [75]. Similarly, to other epigenetic modifications, histone methylation is also a reversible process. However, lysine-specific demethylases (KDMs) action is better understood, whereas arginine demethylation performed by PADI4 and JMJD6 demethylases is considerably less clear [76]. Alterations in histone methylation process have been observed in several cancers, such as gastric carcinoma [77], breast [78] or colon cancer [79], as well as hepatocellular carcinoma [80]. In the case of OSCC, the histone methylation of H3K4 is significantly different in comparison to normal tissues [81]. Furthermore, aberrant methylation of H3K9 carried out by G9a has been observed in HNSCC cells, and may be involved in the lymph node-related metastases and TGF-β-induced EMT [82]. Therefore, histone methylation profiles may be considered as biomarkers of HNSCC detection and metastases. In addition, an elevated level of histone methylation mark at H3K27me3 in HPV(+) HNSCC may, in turn, increase the tumorigenic potential and constitute a HNSCC diagnostic biomarker [83]. Moreover, H3K27me3 regulates the homeobox gene transcription in OSCC and plays a role in neoplastic phenotype of oral keratinocytes [84].

### 2.3. Non-Coding RNA Activity 

Non-coding RNA (ncRNA) can be divided into small (less than 200 nucleotides) and large ncRNA. Small ncRNAs include small nuclear RNA (snoRNA), PIWI-interacting RNA (piRNA), small interfering RNA (siRNA) and microRNA (miRNA). The action of ncRNA is based on the transcriptional and post-transcriptional gene silencing by the specific pairing of bases with target sequences [85]. In this review, we mostly focus on the role of miRNA and lncRNA in HNSCC progression. 

**Figure 2:**
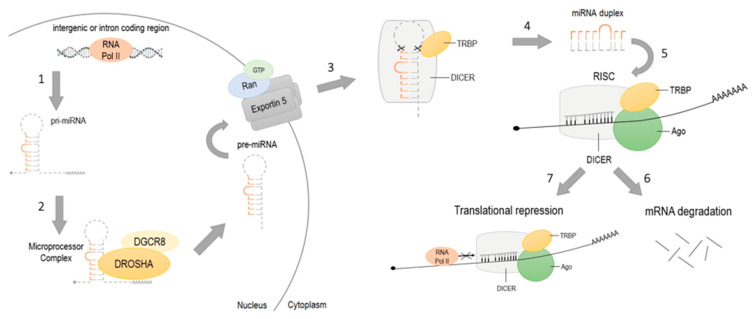
Maturation of miRNA. (1) Transcription of pri-miRNA from intergenic or intron coding regions by RNA polymerase II; (2) recognition of pri-miRNA by DGCR8 and Drosha-cleavage; resulting hairpin- structured pre-miRNA realizing; (3) pre-miRNA transportation from the nucleus to the cytoplasm by Ran/GTP/Exportin 5 complex; (4) pre-miRNA cleavage by Dicer and TAR RNA binding protein (TRBA) to 20–22 nucleotides miRNA; (5) single-stranded miRNA incorporation with Ago and RISC complex connection to target mRNA; gene silencing by (6) translational repression or by (7) mRNA degradation.

MicroRNAs are endogenous small non-coding RNAs regulating the expression of mRNA by interacting with the 3′ untranslated region (3′UTR) of target genes [86]. miRNAs may act as tumor suppressors or as oncogenes (oncomiRs), and play a crucial role in angiogenesis, cell proliferation and apoptosis [87]. Besides, there are several miRNAs influencing gene instability, immune evasion, tumor metastases and chemo- and radioresistance in tumorigenesis [88]. Maturation of miRNA consists of several stages (Figure 2). Transcription of miRNA from intergenic or intron coding region is typically performed by RNA polymerase II [89]. The transcription results in the 5’ capped and 3’ polyadenylated primary transcript (pri-miRNA) which forms hairpin structures. Nuclear protein DGCR8 recognizes pri-miRNA and targets it for Drosha, RNase III-driven cleavage. In fact, about 85 nucleotides long, released hairpin structure, are precursors to miRNA (pre-miRNA). The Ran/GTP/Exportin 5 complex transports pre-miRNA from the nucleus to the cytoplasm where pre-miRNA is processed by RNase III enzyme Dicer and TAR RNA binding protein (TRBA) to double-stranded, miRNA of about 20–22 nucleotides in length [90]. Single-stranded mature miRNA attaches to RNA-induced silencing complex (RISC) and guides RISC to the target mRNA. There are two ways of miRNA gene repression. Firstly, miRNA hybridizes to 3′UTR of the target genes, recruits RISC complex and leads to slitting and degradation of target mRNA. Secondly, miRNA can act as a blocker by connecting to the mRNA and inhibiting its translation [91].

A high-throughput meta-analysis of miRNAs expression shows a long list of miRNAs associated with a poor prognosis, lower survival and metastases in HNSCCs [92]. The dysregulated expression patterns of selected miRNAs were correlated with the clinical stage, lymph node metastases and patient survival, indicating their effectiveness as molecular biomarkers for the HNSCC prognosis [93]. Moreover, the RNA interference mechanism, comprising the action of miRNA and siRNA, has become a valuable research tool for a more comprehensive understanding of the mechanisms regarding HNSCC pathogenesis [94]. Table 2 summarizes the miRNA involved in HNSCC progression.

Following, long non-coding RNAs consist of more than 200 nucleotides and lack protein-coding potential. They are involved in gene expression regulation at both the transcriptional and translational levels, and participate in tumorigenesis and tumor metastases [112,113]. Therefore, lncRNAs expression are promising biomarkers of cancer detection and expansion [114]. LncRNAs are found to play an important role also in HNSCC development. LncRNA ADAMTS9-AS2 expression is significantly upregulated in tongue squamous cell carcinoma (TSCC) of patients with lymph node metastases and follows poor prognostic criteria for advanced disease. The ADAMTS9-AS2 knockdown experiments in TSCC cell lines reduced the cell migration and invasion together with an inhibition of cell growth presented in vitro and in vivo models [115]. Additionally, high expression of lncRNA LINC00460 has been found in HNSCC patients and positively correlated with lymph metastases, pathological differentiation and tumor size [116]. On the other hand, in the case of laryngeal squamous cell cancer (LSCC), high expression of lncRNA MIR31HG is associated with HIF1A and p21 action which leads to an increased cancer cells proliferation [117]. Moreover, lncRNA may act as a tumor suppressor and inhibit tumor growth, e.g., overexpression of lncMX1-215 inhibits H3K27 acetylase resulting in a decreased proliferation of HNSCC cells and a reduced metastatic capacity in vitro and in vivo [118]. Furthermore, overexpression of MYOSLID lncRNA is correlated with upregulation of EMT-related markers, which points to the MYOSLID as a promising controlling biomarker of metastases in HNSCC [119]. Interestingly, Zhang et al. developed a multi-RNA-based model consisting of specific lncRNA, miRNA and mRNA with expression levels correlating with clinicopathological features of HNSCC and predicting survival risk of HNSCC [120]. To summarize, lncRNAs as well as microRNAs expression level has a potential to effectively predict the prognosis and tumorigenesis of HNSCC. 

### 2.4. RNA Methylation 

**Figure 3:**
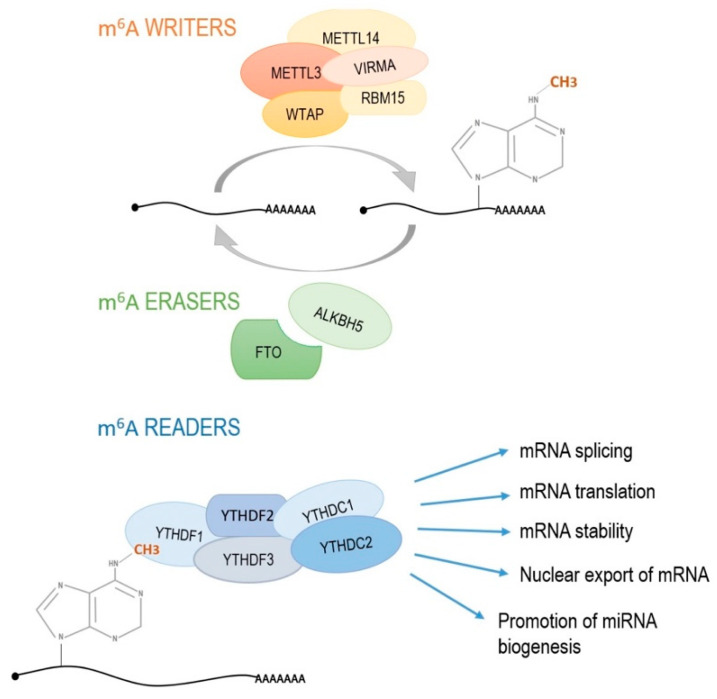
RNA methylation process. Multiprotein complex *writers* are responsible for introducing the methyl group on adenine in position 6. *Erasers* remove the methyl group while *readers* recognize the presence of m^6^A methylation and induce processes, such as mRNA splicing, mRNA translation, mRNA stability, nuclear export of mRNA and miRNA biogenesis. METTL3, methyltransferase like 3; METTL14, methyltransferase like 14; VIRMA, Vir like m^6^A methyltransferase associated; WTAP, WT1-associated protein; RBM15, RNA-binding motif protein 15; FTO, fat mass and obesity-associated protein; ALKBH5, alkB homolog 5; YTHDF1-3 and YTHDC1,2, proteins with YT521-B homology (YTH) domain.

Methylation of adenosine at nitrogen-6 position (m^6^A) in RNA has recently received great attention from cancer researchers. In fact, the m^6^A has been considered as the most prevalent, dynamic and conserved internal transcriptional modification among more than 100 different chemical modifications of RNA [121,122]. Moreover, m^6^A is typically enriched near STOP codon and 3′UTR region containing 5′-RRACH-3′ sequence in which A_3_ becomes N^6^-methylated [123,124]. Reports suggest that this modification has been involved in all stages of RNA processing, including nuclear export, translation modulation to RNA degradation and initiation of miRNA biogenesis [125]. Additionally, m^6^A RNA methylation affects tumor initiation and progression by various mechanisms [126]. RNA methylation related effects are the result of the cooperation of multiprotein complexes known as “writers”, “erasers” and “readers” (Figure 3). The m^6^A methylase complex “writers” consist of:

(1) main catalytic core enzyme which states methyltransferase like 3 (METTL3),
(2) methyltransferase like 14 (METTL14) which structurally positions mRNA for methylation,
(3) WT1-associated protein (WTAP) regulating the recruitment of methyltransferase complex to mRNA targets,
(4) RNA-binding motif protein 15 (RBM15) which is responsible for moving the complex towards the appropriate m^6^A sites and the last “writer” protein,
(5) Vir like m^6^A methyltransferase associated (VIRMA) with uncharacterized molecular function.

The “erasers” complex consists of demethylases FTO (fat mass and obesity-associated protein) and ALKBH5 (alkB homolog 5) which removes the methyl group. The “readers” complex which recognize the presence of the methyl group include YTHDF1-3, YTHDC1 and YTHDC2. These proteins possess YT521-B homology (YTH) domain and participate in the translation, stabilization, splicing and nuclear export of mRNA [127]. YTHDF1 recognizes m6A-modified mRNA and increases the translation efficiency. YTHDF2 recruits the CCR4-NOT deadenylase complex to destabilize and further decay target mRNAs. YTHDC1 is the nuclear m^6^A reader, involved in exon selection during gene splicing. In contrast, YTHDC2 is a putative RNA helicase which cooperates with the meiosis-specific coiled-coil domain-containing protein (MEIOC) and regulates mRNA level during meiosis [128].

Variations in RNA methylation process contribute to tumor growth, progression, invasion and migration of cancer cells in acute myeloid leukemia [129], glioblastoma [130], lung cancer [131], breast cancer [132], liver cancer [133], bladder cancer [134] or pancreatic cancer [135]. In terms of head and neck cancers, disorders in establishing and reading of RNA methylation have been demonstrated in the case of nasopharyngeal carcinoma (NPC) and OSCC. On the basis of the TGCA HNSCC dataset, Zhao et al. demonstrated the significant differential expression of m^6^A RNA methylation regulators between tumor and normal samples [136]. More specifically, Zhang et al. identified an increased level of m^6^A RNA methylation in the ZNF750 gene coding sequence and correlated those changes with ZNF750 lower expression in NPC. The ZNF750 overexpression experiments show cell growth inhibition in NPC in vitro and in vivo models, and indicate the importance of m^6^A RNA methylation in gene expression regulation [21]. Expression of m^6^A machinery elements has also been found to be altered in squamous cell carcinoma. In OSCC patients, METTL3 gene is significantly upregulated in cancerous tissue samples compared to healthy counterparts and these changes correlated with the poor prognosis. The overexpression of METTL3 promoted proliferation, invasion and migration of OSCC cells in vitro, whereas the METTL3 knockdown inhibited the tumor growth in vivo [137]. In addition, m^6^A demethylase ALKBH5 is directly upregulated by DDX3, RNA helicase, which plays an important role in cell proliferation, invasion, and metastases in several kinds of neoplasms. This regulation leads to a decreased m^6^A methylation in FOXM1 and NANOG nascent transcript which contribute to chemoresistance in OSCC [138]. Thus, ALKBH5 has been suggested as a potential target for novel anticancer therapies, due to a direct correlation of its expression with primary HNSCC tumor size [139]. Moreover, the m^6^A modification of lncRNA LNCAROD, mediated by METTL3 and METTL14, enhanced its stability in the HNSCC cells. In in vitro experiments LNCAROD silencing inhibits cell proliferation, mobility, and tumorigenicity, whereas overexpression of LNCAROD in vivo demonstrated opposite results [140]. Considering the crucial role of m^6^A RNA methylation in cell metabolism and unquestionable effects of the disturbances in this process concerning carcinogenesis, RNA methylation as well as RNA methylation-related mechanisms definitely will be discussed in more detail and considered as a candidate for novel, promising HNSCC biomarkers and therapy goals.

## 3. Conclusions

Currently, epigenetic modifications gain more interest in the HNSCC carcinogenesis. Some of them promote cancer formation and progression by controlling the expression machinery. Consequently, the detailed characteristics of the epigenetic changes in HNSCC will ultimately deliver novel, critical prognostic and predictive factors, thus providing the necessary information regarding the treatment and anti-cancer therapies. Moreover, the detailed epigenome-wide profiling may improve both the diagnosis of cancer patients and a target personalized therapy. Although presently, with limited data regarding the mechanism and prognostic value for HNSCC, the role of RNA methylation in carcinogenesis is also worth emphasizing, particularly in terms of a better understanding of the molecular basis of HNSCC and new therapy strategies.

## Figures and Tables

**Table 1:** Hypermethylated gene with diagnostic value identified in head and neck squamous carcinoma (HNSCC).

Gene	Tissue	Type of Study	Diagnostic Significance	References
*ZNF14*, *ZNF160*, *ZNF420*	Tumor and saliva	Meta-analysis confirmed in patient samples	HNSCC detection and surveillance	[40]
*hTERT*	Blood leukocytes	Patient study	HNSCC detection	[41]
*FAM135B*	Tumor	Meta-analysis	Overall survival of HNSCC patients	[42]
*CDKN2A*	Tumor and saliva	Meta-analysis	HNSCC progression and metastases	[43]
*ATM*	Tumor	Patients study	HNSCC detection in early age and early tumor stage	[44]
*MGMT*	Tumor	Meta-analysis	Risk of HNSCC	[45]
*DAPK*	Tumor	Patients study	HNSCC HPV(–) detection in early stage	[46]
*RASSF1A*, *MLH1*, *MGMT*	Tumor	Patients and in vitro study	HNSCC and high proliferative potential of tumor cells detection	[47]
*CTLA4*	Tumor	Patients study	HNSCC detection and surveillance	[48]
*APC*	Tumor	Patients study	Lower number of metastatic lymph nodes	[49]
*CCNA1*, *TIMP3*	Tumor	Patients study	Risk of second primary carcinomas	[50]
*ZIC4*	Tumor	Patients study	Risk of lymph node involvement	[51]
PROM1	Tumor	Meta-analysis	HNSCC detection in early stage and invasion potential	[52]

**Table 2:** Examples of miRNAs involved in the development of head and neck squamous carcinoma (HNSCC).

Process	microRNA	Diagnostic Significance	References
		(Up- or Downregulated)	
**Apoptosis**	miR137	downregulated	[95]
	miR34	upregulated	[96]
	miR17-92	upregulated	[97]
**Gene instability**	miR210	upregulated	[98]
	miR29	downregulated	[99]
**Immune evasion**	miR21	upregulated	[100]
	miR210	downregulated	[101]
**Inflammation**	miR26	downregulated	[102]
	miR218	downregulated	[103]
**Metabolism**	miR26	downregulated	[102]
	miR125b	downregulated	[104]
**Metastases**	miR26	upregulated	[105]
	miR125b	upregulated	[105]
	miR139	downregulated	[106]
	let-7d	upregulated	[107]
	miR200b	upregulated	[108]
	miR218	downregulated	[109]
	miR96	upregulated	[109]
	miR29	downregulated	[99]
	miR200	downregulated	[101]
**Proliferation**	miR21	upregulated	[100]
	miR29	downregulated	[99]
	miR139	downregulated	[106]
	miR155	upregulated	[110]
**Resistance to the radiotherapy and chemotherapy**	miR210	downregulated	[101]
	miR31	upregulated	[111]
	miR125b	downregulated	[104,109]
	miR96	upregulated	[110]
	let-7d	downregulated	[107]
	miR205	upregulated	[107]
	miR96	upregulated	[109]

## Abbreviations

3′UTR	3′ untranslated region
5-caC	5-carboxylcytosine
5-fC	5-formylcytosine
5-hMC	5-hydroxymethylcytosine
5-mC	5-methylcytosine
AID/APOBEC	Activation- induced deaminase/apoplipoprotein B
ALKBH5	AlkB homolog 5
APC	Adenomatous polyposis coli
ATM	Ataxia-telangiectasia-mutated
BER	Base excision repair
BRCA1	Breast cancer type I
CALML5	Calmodulin like 5
CCNA1	Cyclin-A1
CDKN2A	Cyclin Dependent Kinase Inhibitor 2A
CSC	Cancer steam cell
CT	Chemotherapy
ctDNA	Circulating DNA
CTLA4	Cytotoxic T-Lymphocyte Associated Protein 4
DAPK	Death-associated protein kinase
DNMT	DNA methyltrasnferase
EGFR	Epidermal growth factor receptor
EMT	Epithelial-mesenchymal transition
FAM135B	Family with sequence similarity 135 member B
FTO	FTO Alpha-ketoglutarate dependent dioxygenase
GLI1	GLI1 Family Zinc Finger 1
HAT	Histone acetyltransferase
HDAC	Histone deacetylase
HKMT	Histone lysine methyltransferase
HMT	Histone methyltransferase
HNSCC	Head and neck squamous cell carcinoma
HPV	Human papilloma virus
hTERT	Human telomerase reverse transcriptase
JMJD6	Jumonji domain containing 6
KDM	Lysine specific demethylase
LINE-1	Long interspersed nuclear element 1
LSCC	Laryngeal squamous cell carcinoma
LY6D	Lymphocyte antigen 6 family member D
m^6^A	N^6^-methyladenosine
MEIOC	Meiosis specific with coiled-coli domain
METTL	Methyltransferase like
MGMT	O-6-methylguanine-DNA methyltransferase
miRNA	microRNA
MLH1	MutL homolog 1
ncRNA	Non-coding RNA
NF-κB	Nuclear transcription factor-κB
NPC	Nasopharyngeal carcinoma
OSCC	Oral squamous cell carcinoma
PADI4	Peptidyl arginine deiminase 4
piRNA	PIWI-interacting RNA
Pl3K/Akt	Phosphatidylinositol 3-kinase/threonine protein kinase B
PRMT	Histone arginine methyltransferase
PROM1	Prominin 1
RASSF1	Ras association domain family member 1
RB	Retinoblastoma
RBM15	RNA-binding motif protein 15
RISC	RNA-induced silencing complex
RT	Radiotherapy
SAM	S-adenosylomethionine
SAT2	Spermidine/spermine N1-acetyltransferase family member 2
siRNA	Small interfering RNA
SMO	smoothened
snoRNA	Small nuclear RNA
STAT3	Signal transducer and activator of transcription 3
TDG	Thymine-DNA glycosylase
TET	Tet-methylcytosine dioxygenase
TGF-β	Transforming growth factor β
TIMP3	TIMP metallopeptidase inhibitor 3
TRBA	TAR RNA binding protein
TSCC	Tongue squamous cell carcinoma
TSG	Tumor suppressor gene
WTAP	WT1-associated protein
VIRMA	Vir like m^6^A methyltransferase associated
YTH	YT521-B homology domain
ZIC4	Zic family member 4
ZNF	Zinc-finger protein
